# Restoring mitochondrial health after blast-induced traumatic brain injury: modifiable factors and therapeutic opportunities

**DOI:** 10.1038/s44324-026-00111-7

**Published:** 2026-05-13

**Authors:** Cortney J. Laye, W. Brad Hubbard

**Affiliations:** 1 Spinal Cord & Brain Injury Research Center (SCoBIRC), University of Kentucky, Lexington, KY USA; 2 Department of Physiology, University of Kentucky, Lexington, KY USA; 3 Lexington VA Healthcare System, Lexington, KY USA

**Keywords:** Cell biology, Neurology, Neuroscience

## Abstract

Blast-induced traumatic brain injury (blast TBI) causes diffuse neuropathology, blood-brain barrier disruption, and complex neurological sequelae. Mitochondrial dysfunction is increasingly recognized as a contributor to secondary injury cascades and has been associated with bioenergetic impairment, alterations in mitochondrial dynamics, oxidative stress, and apoptotic signaling following blast exposure. Primary data from our recent study demonstrates acute TCA cycle impairment and supports a framework of glycolytic shift in the brain alongside a bottleneck of key TCA cycle intermediates. This review also examines current literature on mitochondrial pathophysiology across neurons, astrocytes, and endothelial cells after blast TBI. We highlight cumulative data detailing disruptions in mitochondrial quality control, including fission–fusion imbalance, and altered mitophagy, as well as bioenergetic dysfunction, calcium dysregulation, enzymatic alterations, and oxidative damage. The influence of lifestyle and environmental modifiers on brain mitochondrial health and how it can alter long-term outcomes after blast TBI are also discussed. We further discuss therapeutic strategies, including mild mitochondrial uncouplers, modulators of mitochondrial dynamics, and mitochondrial transplantation, aimed at preserving mitochondrial integrity and function. Collectively, these findings demonstrate that mitochondrial dysfunction is an important component of blast TBI pathophysiology and supports continued investigation of approaches that integrate modifying factors and therapeutic strategies to improve outcomes after blast TBI.

## Introduction

### Traumatic brain injury (TBI) as a public health challenge

Traumatic brain injury (TBI) is a major global health challenge, with an estimated 27.2 million new cases annually and nearly 49 million individuals living with TBI-related disabilities as of 2019^1^. Clinically, TBIs are classified by severity as mild, moderate, or severe, with mild TBIs accounting for approximately 80% of all cases^2^. Severity is typically defined by the Glasgow Coma Scale Score, duration of altered consciousness, and neuroimaging findings.

However, individuals with similar clinical profiles often experience divergent outcomes, reflecting the heterogeneous nature of an individual’s response to TBI. This variability has prompted support for precision TBI medicine approaches that integrate clinical presentation, molecular biomarkers, neuroimaging, and genetic or environmental modifiers to refine prognostic models and tailor personalized interventions^3–6^. For example, the NIH-NINDS TBI Classification and Nomenclature Initiative has recently introduced the Clinical-Biomarker-Imaging-Modifiers (CBI-M) framework, which emphasizes a multidimensional view of TBI, supporting research and clinical decision making^7^. Yet, while precision frameworks such as CBI-M advance our ability to characterize TBI heterogeneity, there are currently no FDA-approved therapies for TBI, emphasizing the need to translate basic science findings into effective clinical interventions.

### Clinical and epidemiological significance of blast-induced TBI

Beyond severity, TBIs are also classified by mechanism of injury. Closed head injuries, caused by blunt force trauma without skull penetration, account for the vast majority of civilian cases, typically arising from motor vehicle collisions, falls, and sports impacts^8^. In contrast, open head injuries (penetrating TBIs) involve breach of the skull and often have a worse prognosis^9^. Blast-induced TBI (blast TBI), a subtype of close head injury, has garnered particular attention due to its prevalence among military personnel exposed to explosive forces and is termed the “signature wound” of recent military conflicts^10^. An estimated 10–20% of U.S. veterans from Operation Iraqi Freedom and Operation Enduring Freedom have sustained at least one blast-related TBI, with mild blast TBI being the most common subtype^11^. Historically, clinical efforts have emphasized moderate to severe injuries; however, growing evidence indicates that even mild or undocumented blast exposures can result in persistent symptoms. A 2018 study by Clark et al. reported that Veterans with mild TBI and blast exposure show greater frontal cortical thinning and poorer executive functioning compared to those without blast exposure, suggesting that even mild blast-related injuries can lead to lasting cognitive impairments^12^. A more recent study by Dennis et al. found individuals with a history of blast exposure had reduced volume in white matter and subcortical grey matter regions compared to individuals without a history of exposure and were associated with decreased processing speed and working memory^13^.

Clinical management is further complicated by the clinical overlap between post-traumatic stress disorder (PTSD) and blast TBI. Both can present with irritability, sleep disruption, attention deficits, and memory impairment, echoing historical concepts such as “shell shock,” which blurred the line between psychological and physical trauma^14^. Animal models have demonstrated that blast exposure can induce PTSD-related behaviors, such as increased anxiety, enhanced contextual fear conditioning, and altered responses in predatory scent assays^15^. Human studies have also documented personality changes following blast TBI; compared to their pre-injury baseline, blast-exposed individuals were more likely to become aloof or apathetic, suggesting persistent personality consequences^16^.

Despite its prevalence and impact, blast TBI is often considered an “invisible wound,” with mild or absent acute presentation that can minimize the underlying and ongoing cellular dysfunction that can lead to long-term neurological consequences. Beyond persistent post-concussive symptoms, mild blast exposure may increase the long-term risk of developing neurodegenerative diseases. Postmortem analyses of blast-exposed military personnel have revealed hallmark chronic traumatic encephalopathy (CTE) pathology^17^, including multifocal perivascular neurofibrillary and glial tangles immunoreactive for both phosphorylation-independent (tau-460) and phosphorylation-dependent (CP-13) tau epitopes^18^. Complementary population-based studies similarly suggest that older veterans with a history of TBI have a 60% increased risk of developing dementia over nine years, raising concerns about the potential long-term consequences of blast TBI in younger veterans and civilians^19^. These findings underscore the chronic and progressive burden of blast-induced TBI and highlight the need to elucidate the cellular mechanisms that drive progressive neurodegeneration and neuronal dysfunction.

### Biomechanics and neuropathology of blast-induced TBI

A shock wave is a compressive force that travels through a medium at a velocity that exceeds the speed of sound. As it propagates, it rapidly places exposed materials into a shocked state, resulting in a sudden and intense increase in pressure, known as overpressure^20^. The blast pressure-time profile is theoretically described by the Friedlander curve^21^, an idealized waveform characterized by a rapid peak overpressure, followed by a decaying positive phase and a subsequent negative phase. These parameters provide a standardized framework for quantifying blast exposure and can be used to estimate the injury risk based on the standoff distance from the explosion source^22^.

Blast exposure can lead to a spectrum of injury mechanisms classified as primary, secondary, tertiary, and quaternary, with primary blast injuries arising directly from high-pressure shock waves^23^. In the brain, research has shown that blast overpressure induces complex biomechanical effects: (I) direct transmission of the wave and transfer through the skull and brain^24^; (II) abrupt head acceleration caused by uneven, short-lived pressure differences across the skull and brain, which generate shear and inertial forces^24^; and (III) vascular pulse theory, which suggests that blast-induced compression of the thorax or abdomen generates a surge in intravascular pressure that is transmitted to the brain through major blood vessels^25–28^. These forces combine to cause blast TBI that is characterized by primary neuropathology, including axonal shearing, microhemorrhages, and diffuse vascular injury. The cumulative impact of blast forces on the brain results in a myriad of secondary injuries, including disruption of neural and vascular integrity, triggering cascades of neuroinflammation, oxidative stress, blood-brain barrier (BBB) breakdown, and metabolic compromise^29–32^.

Importantly, repetitive or low-level blast exposure, which is common in both combat and training environments, can exert cumulative neurological effects and lead to lasting symptoms and prolonged recovery^33^. Experimental models have reported that single versus repeated blast TBI leads to neurovascular pathological differences in subpopulations of pericytes^34^, nitric oxide signaling^35^, and BBB disruption^36^. The timing between insults is critical; shorter inter-injury intervals exacerbate cognitive impairments and vascular dysfunction, whereas longer recovery periods may mitigate these outcomes, particularly for axonal and motor deficits^37^. Consistent with these findings, our laboratory has demonstrated that mild blast TBI results in a peak in secondary neurovascular injury at 24 h post-injury in male mice^38^, and repeated blast TBI at that time post-injury produces ongoing vascular dysfunction^39^, underscoring the temporal dynamics of vascular vulnerability in the context of repetitive exposure.

Secondary injury cascades after TBI, including vascular alterations, inflammation, neuronal damage and apoptotic signaling^29,40,41^, are often downstream of mitochondrial dysfunction. Mitochondria regulate energy metabolism, redox balance, and apoptotic signaling, making them key regulators of TBI pathology. Our review focuses specifically on mitochondrial and metabolic dysfunction in blast TBI as a hallmark mechanism driving secondary injury. Mitochondrial impairment is particularly detrimental in cells with high energy demand, such as neurons, astrocytes, and cerebrovascular endothelial cells, where synaptic activity, neurovascular coupling, and BBB integrity can be disrupted. This review, therefore, focuses on mitochondrial mechanisms to illuminate how these organelles contribute to the ongoing effects of blast TBI.

## Metabolic compromise and mitochondrial pathophysiology in blast TBI

The human brain has comparatively high energy demands, consuming 20% of the body’s oxygen despite only 2% of the body’s weight^42^, making it particularly sensitive to metabolic challenge and vulnerable to mitochondrial dysfunction. Human PET imaging studies of blast-exposed individuals reveal hypometabolism within the cerebellum, thalamus, pons, and medial temporal lobe, with cerebellar deficits being the most significant^43^. Similar findings in a study with 12 Iraq combat veterans exposed to blast TBI^44^, within this cohort, blast-exposed individuals exhibited subtle impairments in complex information processing, including mild reductions in verbal fluency, processing speed, attention, and working memory. Another human study found hypometabolism in the right superior parietal region and suggested greater post-concussive sequelae, such as impaired attentional control, compared to blunt mild TBI^45^. Lastly, a study of military breachers and range staff identified a distinct metabolomic signature including reductions in several metabolites related to energy metabolism (acetic acid, formate, creatine, acetone, methanol) and the excitatory amino acid glutamic acid in personnel experiencing post-concussive symptoms and poorer overall health following repeated low-level exposure^46^. Interestingly, three metabolites: acetic acid, creatine and methanol negatively correlated with the Rivermead post-concussion symptoms questionnaire^46^. These findings suggest that blast-related metabolic disturbances extend beyond focal lesions and may reflect altered network-level bioenergetics; however, PET-based hypometabolism alone cannot distinguish between reduced metabolic demand and primary mitochondrial impairment. Together, these observations provide a rationale for mechanistic studies in preclinical models to better define the biological mechanisms underlying metabolic alterations after blast TBI.

Mitochondria support neuronal viability by regulating oxidative metabolism, redox balance, calcium homeostasis, and apoptotic signaling^47^. Under physiological stress, these organelles engage quality control mechanisms, such as biogenesis, dynamics (fission and fusion), and mitophagy, to preserve their integrity and sustain cellular homeostasis^48,49^. Based on accumulating evidence over the past two decades, blast exposure disrupts these safeguards, leading to the accumulation of damaged mitochondria, redox imbalance, and apoptotic priming^50–59^. Experimental models across blast TBI have been reported to impair mitochondrial morphology and respiration, elevate reactive oxygen species (ROS) levels, and compromise neurons, astrocytes, and cerebrovascular endothelial cells.

Among TBI subtypes, our current knowledge represents mitochondrial dysfunction as a conserved secondary injury pathway encompassing impaired bioenergetics, oxidative stress, disrupted mitochondrial dynamics and apoptotic signaling. Similar to impact and rotational TBI, blast TBI triggers secondary cascades of excitotoxicity, oxidative stress, and apoptotic signaling, reflecting a conserved mitochondrial vulnerability across injury subtypes. Foundational work by Opii et al., using a preclinical blunt impact TBI model, identified mitochondria as key drivers of secondary injury cascades, where calcium dysregulation and ROS accumulation, coupled with reductions in pyruvate dehydrogenase and oxidative phosphorylation, underlie cellular energy failure^60^. Comparable defects have been observed in blast models, indicating shared metabolic vulnerability across TBI types^50,52^. Direct comparative studies remain limited between impact and blast TBI models, and blast exposure presents a clinical challenge because of added complexity due to systemic exposure^61^. Nevertheless, blast TBI results in similar mechanisms of mitochondrial dysfunction as impact TBI, although diffuse blast TBI likely influences the timing and spatial distribution of mitochondrial deficits. Our review focuses on the studies, to date, that highlight mitochondrial impairments and the experimental details for all blast TBI studies in the following sections are presented in Table 1.

**Table 1:** Summary of preclinical and in vitro studies investigating mitochondrial dysfunction following blast exposure

Study (Year)	Animal model	Sex	Age	Blast exposure (peak pressure)	Positive impulse	Blast device	# of Blast exposures	Time point(s) post-injury	Primary mitochondrial outcome metrics
Schmitt et al.^58^	Human brain microvascular cells	N/A	N/A	~23 PSI	N/A	Compressed air driven shock tube	Single	0.5 h– 48 h	Mitochondrial genes expression, ROS, calcium influx, apoptosis
Guilhaume-Correa et al.^57^	Primary astrocytes cell culture	N/A	N/A	~17–20 PSI	~7.4 PSI-ms	High-rate overpressure simulator	Single	4 h–3 days	Mitochondrial morphology, protein levels of Drp1
	Sprague–Dawley rats	M	10 weeks	~17–20 PSI	~15.6 PSI-ms	Advanced blast simulator	Single	4 h–7 days	Protein levels of Drp1
Hubbard et al.^51^	Sprague–Dawley rats	M	8 weeks	~11 PSI	~37 PSI-ms	Helium gas shock tube	2× (48 h)	2 days	Synaptic vs. non-synaptic, mitochondrial function analysis
Arun et al.^50^	C57BL/6J mice	M	8–10 weeks	~21 PSI	N/A	Compressed air driven shock tube	Single and 3× (1 and 30 min intervals)	1 h–24 h	Mitochondrial GOT2 activity, pyruvate dehydrogenase levels
Xu et al.^56^	New Zealand rabbits	M & F	Adult	~332 PSI	~2.4 PSI-ms	Explosion frame of TNT	Single	1 h–14 Days	Caspase-3, Cytochrome C, Bax, Bcl-2
Wang et al.^55^	C57BL/6J mice	M	8–10 weeks	~13.4 PSI	N/A	Compressed air driven shock tube	3x (1 and 30 min intervals)	2 h–7 Days	DNA fragmentation, membrane potential, Cytochrome C, Caspase-3
Okonkwo et al.^52^	Sprague–Dawley rats	M	Adult	~20 PSI	N/A	Advanced blast simulator	3x (15 min interval)	0–3 h	Blood plasma levels of lactate, PDH, Complex I, and IV activity
Song et al.^54^	C57BL/6J Mice	M	8 weeks	~6.76 PSI	~8.7 PSI-ms	Open field C4 explosives	Single	7 days and 30 days	Ultrastructural mitochondrial morphology
Song et al.^53^	C57BL/6J mice	M	8 weeks	~6.76 PSI	~8.7 PSI-ms	Open field C4 explosives	Single	3 h–30 days	Fission, fusion, complex I–IV, mitophagy protein levels
Laye and Hubbard,^147^	C57BL/6J-Tyrc-2J	M	8 weeks	~11 PSI	~37 PSI-ms	Helium gas shock tube	2× (24 h)	1 day	Untargeted metabolomics
									LC-MS
Kumari et al.^69^	Sprague–Dawley rats	F	10–12 weeks	~14.5 PSI	N/A	Helium gas shock tube	3× (1 min, 30 min and 24 h)	1 day	Untargeted metabolites
									H NMR
Rana et al.^59^	Sprague–Dawley rats	M	10 weeks	~26 PSI	N/A	Helium gas shock tube	Single	1 day–7 days	Metabolomics, oxidative stress, mitochondrial metabolism, NOX

Peak static overpressure, positive duration, and positive impulse values are reported where available. For Xu et al.^56^, positive impulse values were obtained using the blast conversion model described in the original study and calculated via the Kingery–Bulmash Blast Parameter Calculator (UN SaferGuard). For studies in which a positive impulse was not reported, the impulse was estimated using the Friedlander equation with decay constant *b* = 1, which assumes a canonical free-field exponential decay.

*M* male, *F* female, *N*/*A* not available.

### Acute metabolic and enzymatic disruptions in blast TBI

Blast TBI disrupts early metabolic entry points into the mitochondria, with pyruvate dehydrogenase (PDH) serving as a point of vulnerability^50,52^. PDH catalyzes the conversion of pyruvate to acetyl-CoA, making it a central regulator of tricarboxylic acid (TCA) cycle activity and oxidative phosphorylation^62^. Arun et al. reported that blast TBI induces acute (6–24 h) PDH inhibition in the cerebral cortex^50^. PDH deficiency has been reported in several neurodegenerative disorders leading to metabolic failure, secondary lactic acidosis and production of ROS^63^.

**Fig. 1:**
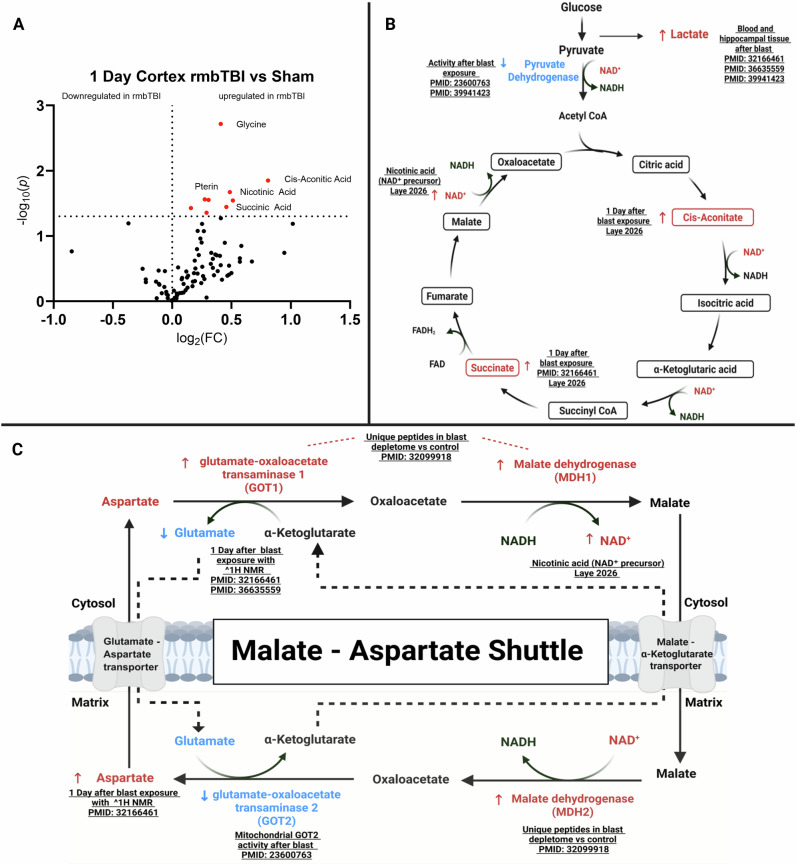
Acute metabolic and mitochondrial pathway alterations following blast inducedtraumatic brain injury. **A** Volcano plot showing differential metabolite levels in the cortex of repeated mild blast TBI versus sham animals at 1 day post-blast TBI. Red-colored data points indicate significantly upregulated metabolites, including glycine, pterin, nicotinic acid, succinic acid, and cis-aconitic acid. At 1 d post-blast TBI, mice were decapitated, and the cortex was rapidly dissected and flash frozen in liquid nitrogen. Cortical samples were processed and analyzed using LC-MS to resolve metabolomic profiles. **B** Simplified schematic of TCA cycle alterations after blast TBI. Metabolites with increased levels post-blast TBI are highlighted in red; decreased activity or downregulated enzymes are indicated in blue. **C** Schematic of the malate-aspartate shuttle with mitochondrial and cytosolic compartments. Blast-induced alterations in enzyme expression or activity are shown in red (upregulated) and blue (downregulated). Dashed lines indicate transport of intermediates across the mitochondrial membrane. References indicate the source of supporting experimental data. Created using BioRender.

Moving into the TCA cycle, our recent study shows altered metabolomics at 1 day post-blast TBI, including accumulation of succinic acid and cis aconitic acid, indicating TCA cycle stalling (Fig. 1A). Elevated nicotinic acid suggests activation of NAD salvage pathways in response to mitochondrial redox stress. Results from our study, paired with others in blast TBI support a glycolytic shift, signified by increased lactate, alongside a bottleneck of key TCA cycle intermediates (Fig. 1B). Consistent with prior reports linking elevated myo-inositol and glycine to gliosis and cognitive impairment^64^, we similarly observed increased glycine following blast exposure, suggesting overlapping glial and metabolic responses in blast TBI. Additional evidence of mitochondrial disruption is provided by succinate accumulation, particularly in the hippocampus, a region vulnerable to hypoxia and oxidative stress. Hippocampal succinate accumulation occurs after blast TBI, implicating reduced succinate dehydrogenase (SDH, Complex II) activity^59^. As complex II couples the TCA cycle to the electron transport chain, its impairment not only diminishes ATP production but also drives reverse electron transport at complex I, amplifying ROS production^65^. Consistent with this mechanism, hippocampal neurons displayed the largest increases in NADPH oxidases (NOX 1 and NOX2) compared with astrocytes and microglia, highlighting the synergistic interactions between mitochondrial dysfunction and extramitochondrial ROS production^59^. This feedback loop accelerates oxidative damage and magnifies neuronal vulnerability to blast TBI.

Yudkoff and colleagues showed that amino acids can function as an alternative energy source, supporting cerebral ATP production via mini-citric acid cycle^66^. In the brain, particularly glutamate is rapidly mobilized to meet acute energetic demands. Consistent with this metabolic adaptation, blast exposure appears to compromise the malate-aspartate shuttle, a critical pathway for transferring cytosolic reducing equivalents into the mitochondria. Experimental blast models are reported to reduce cortical ATP levels and suppress activity of mitochondrial glutamate–oxaloacetate transaminase (GOT2), also known as mitochondrial aspartate aminotransferase (mAST), which catalyzes the conversion of glutamate and oxaloacetate to alpha-ketoglutarate and aspartate^50^. Within this pathway, GOT2 links amino acid metabolism to the TCA cycle and NADH shuttle via the malate-aspartate shuttle^67^. Complementing these findings, proteomic analyses after blast TBI identified unique peptides in cytosolic (MDH1) and mitochondrial malate dehydrogenase (MDH2), along with cytoplasmic aspartate aminotransferase (GOT1) following depletion of abundant proteins, indicating possible alteration of the mitochondrial and cytosolic arm of the MAS^68^. Together, these observations support compartmentalized uncoupling of the shuttle, thereby restricting NADH-linked complex I mitochondrial respiration and contributing to TCA cycle dysfunction following blast TBI (Fig. 1C).

Metabolomic profiling by Rana et al. using ^1^H NMR spectroscopy revealed that blast TBI elevates cortical aspartate and reduces glutamate 1 day after blast, a pattern consistent with shuttle failure and transaminase inhibition^59^. Although these measurements could not distinguish between cytosolic (GOT1) and mitochondrial (GOT2) isoforms, the shifts aligned with aspartate aminotransferase (AST) dysfunction and further suggested impaired α-ketoglutarate availability, disrupted glutamatergic neurotransmission, and compromised astrocyte–neuron metabolic coupling. Together, these metabolic signatures reinforce a model in which blast-induced disruption of amino acid–dependent redox shuttling limits mitochondrial oxidative metabolism while simultaneously perturbing excitatory neurotransmitter homeostasis. Collectively, these studies highlight AST/GOT dysfunction as a possible mechanistic link between mitochondrial bioenergetic failure and synaptic-level network disruption in blast TBI.

### Mitochondrial dysfunction across the neurovascular unit

Disruptions in mitochondrial enzymes and metabolic shuttles give rise to characteristic neurometabolic signatures. Kumera et al. reported reductions in N-acetyl aspartate (NAA) and glutamate levels, alongside lactate accumulation, reflecting widespread impairments in mitochondrial oxidative metabolism^69^. NAA, a neuron-specific metabolite synthesized in the mitochondria, is a marker of neuronal integrity and neuron–glia metabolic coupling; its reduction indicates compromised bioenergetic support^70^. Translational studies using magnetic resonance spectroscopy in humans similarly revealed reduced NAA/choline and NAA/Creatinine ratios in the hippocampus, which correlated with deficits in visual memory performance, underscoring the possible clinical impact of these mitochondrial metabolic disruptions^71^. Song et al. observed marked neuronal mitochondria abnormalities, including cristae disorganization, matrix swelling and vacuole formation in the hippocampus and cortex, with hippocampal vulnerability peaking at 7 days and cortical disruption persisting up to 30 days^54^.

Our group further characterized synaptic mitochondrial vulnerability following repeated mild-blast TBI. Respiration supported by complex I–linked substrates reflects electron entry through NADH-generating pathways, whereas complex II–linked substrate conditions assess electron flow via FADH₂-dependent pathways. Together provide a functional readout of oxidative phosphorylation capacity and mitochondrial energy production. In the amygdala–entorhinal–parietal network, we found a marked reduction in maximal electron transport capacity, reflecting a diminished ability to meet increased energetic demand under stress conditions. In contrast, the prefrontal cortex showed impairments in both ATP-linked respiration (reflecting energy production under physiologic conditions) and maximal respiratory capacity, indicating a broader deficit affecting both basal energy generation and reserve bioenergetic capacity^51^. No significant changes were detected in the hippocampal synaptic mitochondria, highlighting possible region- and compartment-specific susceptibility in synaptic vs non synaptic mitochondrial across blast models. These functional deficits were accompanied by oxidative stress; glia-enriched mitochondria exhibited elevated lipid peroxidation and nitration markers, including 4-hydroxy-2-nonenal (HNE) in the prefrontal cortex and both HNE and 3-nitrotyrosine in the hippocampus. These neuronal deficits seem to be paralleled by mitochondrial dysfunction in astrocytes, which normally buffer oxidative stress and support metabolic coupling.

In primary astrocyte cultures, mechanical blast induced acute fragmentation and loss of networked structures within 4 h, partially recovering by 3 days post-injury via mitochondrial network analysis. These sustained structural compromises likely exacerbate oxidative stress, impair ATP production, and contribute to chronic bioenergetic defects^57^. Mechanistically, Guillaume-Correa et al. identified increased phosphorylation of Drp1 at Ser616 in vitro and in vivo, a modification associated with pathological fission activity^57^. These astrocyte-related changes in mitochondrial dynamics are echoed by similar changes in neuron-associated mitochondrial dynamics.

Beyond the effects of blast TBI on mitochondrial function in neurons and astrocytes, blast TBI-induced alterations in the mitochondrial health of blood vessels have also been reported. Schmitt et al., in vitro endothelial blast model, observed pronounced mitochondrial impairment in human brain microvascular endothelial cells (HBMVECs). Early overexpression of mitochondrial genes encoding core complex I subunits (NDUFV1 and ND1) suggested dysregulated NADH oxidation, while ROS increased by 47% and intracellular calcium levels by 11% within 3 h post injury, rising to a total of 168% by 6 h^58^. This endothelial stress compromises blood-brain barrier (BBB) integrity, propagates neuroinflammation, and exacerbates secondary neuronal injuries. In vitro blast exposure led to a ~40% decline in HBMVEC viability and a fourfold increase in caspase-3 activity, with apoptosis accounting for the majority of endothelial cell death. Collectively, these findings link early mitochondrial failure and oxidative stress to downstream vascular injury and intrinsic apoptosis.

Under physiological conditions, our lab has found that astrocytes transfer mitochondria to endothelial cells and pericytes to support vascular energy metabolism^72^. Astrocytes have also been shown to transfer mitochondria to neurons under cellular stress^73,74^. However, following a blast, metabolic stress and ROS accumulation may impair this transfer, contributing to endothelial mitochondrial dysfunction, BBB disruption, and secondary neuronal injury. Thus, interventions that preserve astrocytic mitochondrial integrity may have dual benefits: directly protecting neurons and sustaining endothelial resilience.

### Mitochondrial fission–fusion imbalance in blast TBI

Mitochondria maintain their functional integrity through a balance of dynamics, including fission and fusion, orchestrated by conserved GTPases. Fusion proteins, primarily mediated by mitofusin-1 (MFN1), mitofusin-2 (MFN2), and optic atrophy protein-1 (OPA1), support mitochondrial elongation and content mixing, and efficient oxidative phosphorylation under stress^75^. Fission, driven mainly by dynamin-related protein-1 (Drp1) and mitochondrial fission 1 protein (FIS1), segregates damaged mitochondrial segments for subsequent clearance by mitophagy^75^. We also note that preclinical studies have demonstrated that traumatic brain injury increases mitochondrial fission, and that modulating fission–fusion dynamics can influence functional recovery, highlighting the translational relevance of these processes^76^.

Blast overpressure disrupts this homeostatic balance, possibly biasing it toward excessive fission. Low-level blast exposure demonstrated that MFN2 and Fis1 were significantly reduced at 7- and 30-days post-injury, while Drp1 and OPA1 decreased as early as 3 h and persisted through 30 days^53^. Despite Drp1 downregulation, phosphorylation at ser616 was elevated in astrocytes, enhancing fission activity, which may exacerbate blood- brain barrier instability and disrupt neurovascular coupling^57^. Parkin levels were also decreased at 7- and 30-days post injury, suggesting impaired mitophagy and clearance of damaged mitochondria^53^. These molecular alterations may correlate with transmission electron microscopy findings of fragmented and swollen mitochondria in cortical and hippocampal neurons^54^. Although most studies focus on neurons, evidence indicates that astrocytic and vascular mitochondria may also exhibit dysregulated dynamics, which could amplify secondary injury cascades.

### Electron transport chain dysfunction and apoptosis in blast TBI

Blast-induced mitochondrial dysfunction extends beyond enzymatic and neurovascular disruptions and is associated with suppressing the electron transport chain (ETC) and contributing to oxidative stress and bioenergetic failure. In an open-field low-level blast model, Song et al. reported widespread downregulation of ETC subunits, including Complex II (SDHB), Complex III (UQCRC2), Complex IV (MTCO1), and Complex V (ATP5PA), detectable within 3 h post-injury and persisting up to 30 days^53^.

As mitochondrial failure advances, apoptosis is engaged, marking the point of no return for cellular survival. Wang et al., using the comet assay, observed a time-dependent increase in DNA fragmentation starting at 2 h and peaking at 24 h post-injury in the cerebellum and cortex^55^. Although DNA fragmentation may reflect a direct mechanical effect of the blast wave, the concurrent activation of mitochondrial pathways suggests that intrinsic apoptosis may involve more than secondary injury, potentially representing a coordinated response to bioenergetic stress. Blast TBI studies have also reported hallmarks of mitochondria-mediated apoptosis, including loss of mitochondrial membrane potential, cytochrome c release, and caspase-3 activation, highlighting the mechanistic link between mitochondrial bioenergetic failure and programmed cell death^55^. Complementing these findings, Xu et al. demonstrated increased cleaved caspase-3, caspase-9, and cytochrome c expression in brain tissue after blast TBI as early as 1 h post-blast and peaking at 12 h^56^. Collectively, these studies suggest that early mitochondrial-mediated apoptosis contributes to injury following blast exposure.

### Biomarkers of metabolic and mitochondrial alterations after blast TBI

Beyond central nervous system assessments, blast-induced metabolic dysregulation in tissue-level disruptions is also observed in peripheral biofluids. Okonkwo et al. employed a rapid, blood-based lateral flow dipstick assay to quantify PDH and electron transport chain complex activity, revealing acute reductions in PDH and Complex I/IV activity within 1–3 h of repetitive blast exposure, accompanied by elevated plasma lactate levels^52^. Complementing these findings, recent LC–MS-based plasma metabolomics demonstrated that mild blast exposure significantly elevated fructose and reduced succinic acid 1- and 7-days post injury^77^. Interestingly, one study with military breachers and ranger staff found low levels of glutamic acid in plasma^46^ and another study at Urban Mobility breacher course found decreased glutamic acid in urine of exposed individuals^78^. Collectively, these studies suggest that plasma metabolites may serve as accessible biomarkers of blast-induced mitochondrial dysfunction and provide a translational link between preclinical mechanistic studies and potential human diagnostics.

## Systemic and environmental modifiers of mitochondrial dysfunction after blast TBI

While the acute mitochondrial disruptions described in the above sections unfold within minutes to days after blast exposure, the long-term trajectory of mitochondrial health in the brain is not fully determined by the initial injury severity. Recovery is shaped by systemic and environmental factors, particularly those relevant to military personnel and Veterans, which can hinder mitochondrial recovery or exacerbate chronic dysfunction. Within the context of the CBI-M framework, these modifiers can be viewed as secondary drivers that converge on shared mitochondrial pathways, thereby shaping the chronic trajectory of dysfunction. These modifiers could create additive or synergistic effects on oxidative stress, mitochondrial dynamics, and bioenergetics. Recognizing how these factors influence recovery is critical for identifying therapeutic windows and developing targeted interventions.

There is recognition that blast TBI disproportionately affects military personnel, who are challenged by unique factors during active service, such as sleep disruption and societal reintegration. As such, there is a need to account for these unique challenges in preclinical blast exposure models to make military-related research more clinically relevant. To increase translational relevance, preclinical blast models are beginning to incorporate such modifiers to evaluate their additive impact on mitochondrial health and neurological outcomes^79–81^. Our group and others have found that mitochondrial function is a reliable surrogate for both cellular function and neurological performance^82–84^. Thus, understanding the effect that lifestyle modifiers can shape brain mitochondrial function is critical to brain recovery or decline after blast TBI.

### Sleep and circadian rhythm effects

Veterans with blast-related mTBI exhibit reduced regional cerebral glucose metabolism during REM sleep within limbic and paralimbic regions, including the amygdala, hippocampus, thalamus, and brainstem, compared with non–blast-exposed controls^85^. REM sleep supports synaptic remodeling, emotional regulation, and metabolic restoration. Because of this REM-specific hypometabolism, it suggests that blast TBI disrupts sleep-dependent neuro recovery processes. Importantly, sleep disturbance correlated with poorer cognitive outcomes and PTSD symptom severity, underscoring sleep dysfunction as a clinically relevant modifier of blast-related neurological sequelae^86^.

Preclinical studies further demonstrate that blast exposure perturbs circadian regulatory systems. In a rat model of blast TBI, plasma and cerebrospinal fluid melatonin levels were reduced, coinciding with sleep/wake disturbances, and implicating pineal gland dysfunction as a potential mediator of circadian disruption^87^. Consistent with this, blast altered core clock gene expression in both the pineal gland and hypothalamus, including increased Bmal1 and reduced Per1, Per2, and Cry2 at 24 h post-injury, suggesting acute disturbances in circadian rhythm signaling^88^. Circadian clock signaling is thought to influence mitochondrial processes such as oxidative phosphorylation and ROS^89^, thus, circadian misalignment following blast exposure may exacerbate mitochondrial vulnerability and disrupt temporal coordination of energy production and repair pathways. Together, these findings position sleep and circadian disturbance as biologically meaningful modifiers that may amplify mitochondrial dysfunction and impair recovery following blast TBI.

### Alcohol use disorder

An important consideration for factors after blast TBI is the potential deleterious effect of alcohol on brain mitochondria. Several studies have modeled alcohol consumption following preclinical blast TBI. One study found that pre-injury ethanol consumption contributed to exacerbated fear extinction alterations after blast TBI in male and female mice^79^. Post-trauma alcohol consumption following mild blast TBI showed early anxiolytic and short-term memory benefits, but prolonged drinking impaired long-term memory and increased oxidative stress, suggesting a pathological synergy between brain injury and alcohol exposure^80^.

Clinical analysis of AUDIT-C scores in Iraq/Afghanistan Veterans revealed that individuals with blast-mild TBI with loss of consciousness shifted from “frequent” to “risky” drinking profiles compared to blast mild TBI with only altered consciousness^90^. Consistent with this finding, repetitive blast exposure in mice revealed an increased sensitivity to ethanol, evidenced by greater locomotor stimulation at low doses and an earlier surge in voluntary alcohol consumption^90^. These parallel findings across species indicate that repetitive blast may promote risky alcohol- related behaviors. Given that ethanol metabolism may increase mitochondrial reactive oxygen species production and uses NAD in the oxidative pathways of alcohol metabolism^91^, the convergence of blast-induced mitochondrial dysfunction with alcohol-related oxidative stress may contribute to neurodegenerative processes and cognitive decline.

### Psychological stress and environmental resilience

Psychological resilience also emerges as a clinically relevant modifier of blast outcomes. In Iraq and Afghanistan combat Veterans, Martindale et al. identified distress tolerance (DT) as a significant moderator of psychiatric and functional impairment following blast exposure, mild TBI, and PTSD^92^. Higher DT was associated with improved sleep quality, reduced posttraumatic and depressive symptom severity, and better overall quality of life, independent of blast severity or TBI history^92^. Experimental models further support the role of environmental factors in blast recovery. Rodents exposed to whole body blast studies have examined the effects of long-term housing in enriched environments featuring social interaction, cognitive stimulation, and physical activity (e.g., running wheels, tunnels, and balls)^81^. Enrichment partially restored memory function, although anxiety-like behaviors were largely unaffected, suggesting that environmental stimulation may support functional recovery and merit further investigation^81^.

### Cognitive reserve and individual variability

Individual variability in functional outcomes following blast exposure may reflect differences in cognitive reserve. Cognitive reserve refers to the brain’s capacity to maintain function through flexible recruitment of neural networks or alternative cognitive strategies, even in the presence of injury or pathology^93^. In the context of blast TBI, individuals with higher cognitive reserve may be better able to compensate for mitochondrial dysfunction, synaptic loss, or other cellular insults. Considering cognitive reserve as a moderating factor could help explain the wide spectrum of clinical outcomes observed following similar levels of blast exposure.

### Summary of modifiers of mitochondrial dysfunction after blast TBI

**Fig. 2:**
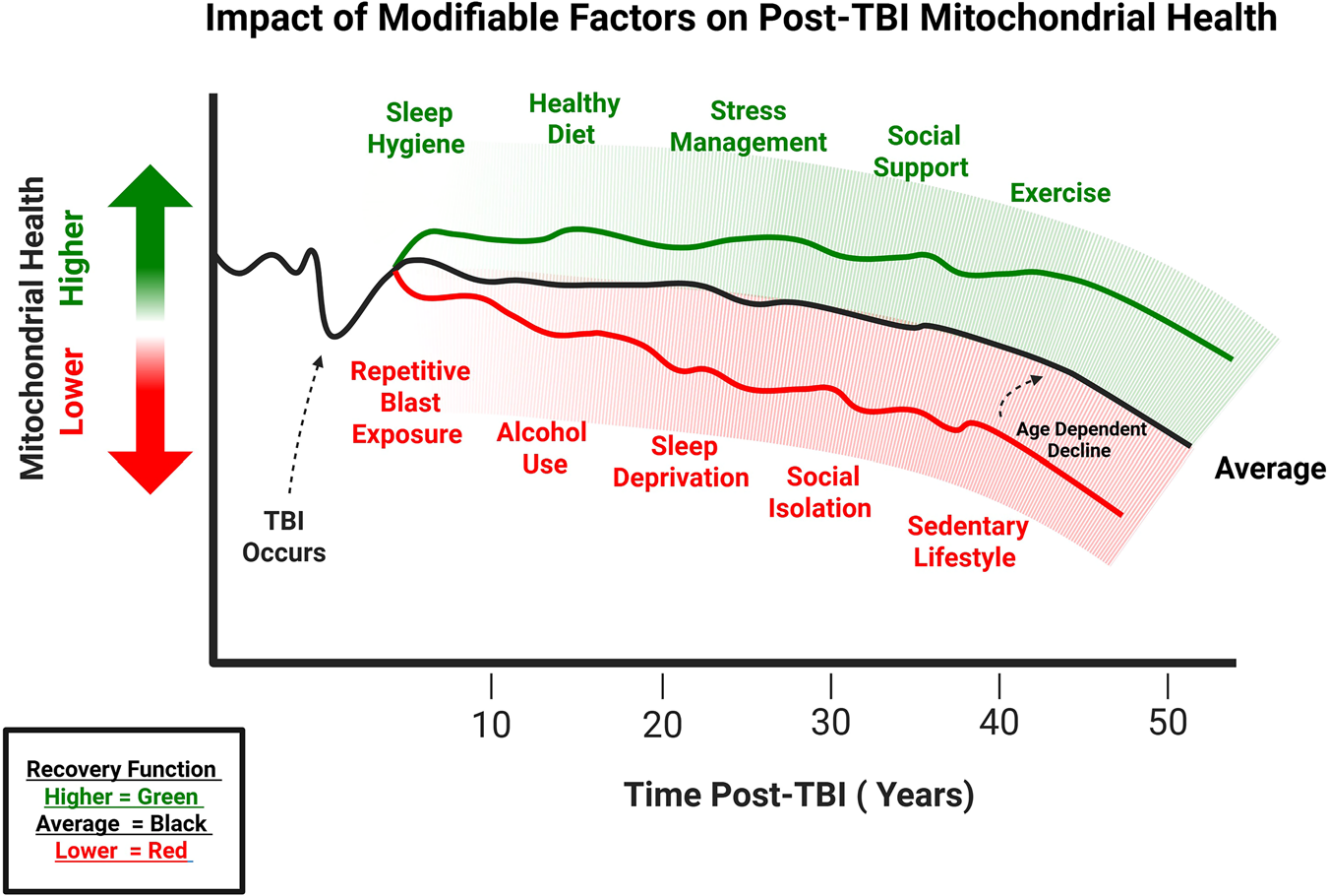
Example of the influence of modifiers on lifetime brain mitochondrial health after blast TBI. Factors that negatively impact mitochondrial health, such as repetitive blast exposure and chronic alcohol use, are shown on the lower end of the spectrum, whereas positive modifiers, including exercise and social support, are depicted on the higher end. The average line represents the hypothetical time course of mitochondrial health in the brain, highlighting how cumulative modifiers can shift overall mitochondrial health. Created using BioRender.

Environmental and systemic factors, including sleep, circadian rhythms, alcohol use, psychological stress, metabolic status, physical activity, and social context, modulate the trajectory of mitochondrial recovery or dysfunction after blast TBI (see Fig. 2). Social factors, such as social isolation or limited support networks, can impair ATP production and apoptosis regulation and exacerbate ROS, impairing mitochondrial function^94^. Dietary habits can influence mitochondrial function, with high-fat or nutrient-poor diets exacerbating mitochondrial dysfunction and risk for metabolic disease, while interventions such as ketogenic diets, fasting, or Mediterranean-style eating can enhance mitochondrial resilience, modulate cellular bioenergetics, and support long-term health outcomes^95^. These modifiers converge on the same pathways disrupted acutely by blast (ETC, fission/fusion balance, ROS production, bioenergetics), producing additive or synergistic effects. Recognizing these interactions highlights opportunities for holistic interventions to support mitochondrial resilience, limit secondary injury, and improve long-term neurological outcomes.

## Therapeutic strategies targeting mitochondrial dysfunction in blast TBI

Mitochondrial dysfunction plays a central role in both acute and chronic pathophysiology following blast TBI, modulating risk and driving metabolic failure, oxidative stress, and apoptotic signaling. Therapeutic strategies aimed at restoring mitochondrial homeostasis have therefore garnered significant interest. Currently, no FDA-approved treatments exist for blast TBI; however, preclinical studies highlight a variety of promising interventions targeting oxidative stress, bioenergetic failure, mitochondrial dynamics, and apoptotic pathways, with the potential to mitigate neuronal injury and improve functional outcomes. In the following sections, we highlight potential therapeutic strategies that could be utilized to target mitochondrial dysfunction after blast TBI.

### Targeting apoptotic pathways

Mitochondria act as central regulators of cell fate after TBI, and although mitochondrial permeability transition pore (mPTP) opening has not been directly shown in blast TBI, Wang et al. reported elevated cytochrome c levels, suggesting calcium-mediated mitochondrial stress and activation of apoptotic pathways^55^. Therapeutic interventions have therefore focused on blocking mPTP formation, with cyclosporin A (CsA), an FDA-approved immunosuppressant, repurposed for its ability to bind cyclophilin D and prevent pore opening. In controlled cortical impact models, CsA preserved mitochondrial membrane potential^96^ and improved synaptic and non-synaptic mitochondrial energetics^97^, while genetic ablation of cyclophilin D reduced mPTP opening and improved mitochondrial integrity and functional outcomes^98^. CsA stabilizes mitochondrial integrity by preventing calcium-induced apoptosis and is likely more effective early after TBI due to excitotoxic effects. Importantly, this strategy has been translated to the clinic: the Copenhagen Head Injury Ciclosporin Study demonstrated feasibility, safety, and biomarker modulation in severe TBI patients^99^. Although direct studies in blast TBI remain limited, Wachtler et al. recently emphasized the heterogeneity of calcium dysregulation following blast exposure and its potential as a therapeutic target^100^.

### Mild mitochondrial uncoupling

Mitochondrial uncoupling is an endogenous process that is regulated by uncoupling proteins^101^ but this process can be supplemented with exogenous uncouplers that offer a targeted approach to modulate bioenergetics, reduce ROS production, and restore mitochondrial function after injury^102^. Mitochondrial uncoupling occurs when protons re-enter the mitochondrial matrix independent of ATP synthase, partially dissipating the proton gradient. This allows the electron transport chain (ETC) to continue operating efficiently without over-reducing its components, which in turn reduces the generation of ROS. By limiting ROS production and preventing excessive mitochondrial calcium accumulation, mild mitochondrial uncoupling can protect mitochondria and cells from oxidative stress and calcium-induced damage. As opposed to traditional antioxidants, such as MitoQ^103^, mitochondrial uncoupling reduces free radicals at the source. MP201, a prodrug of 2,4-dinitrophenol (DNP), is a mild mitochondrial uncoupling agent with a larger safety index compared to classical uncouplers such as FCCP or CCCP^104^. Consistent with this, micro-dose DNP may be used as a potential novel treatment for Amyotrophic Lateral Sclerosis, as a recent study reports lower mitochondrial membrane potential compared to FCCP and reduced mitochondrial calcium uptake^105^. This improved safety profile has facilitated its investigation in a range of CNS disorders, including Alzheimer’s disease, epilepsy, Huntington’s disease, and optic neuritis, with promising neuroprotective outcomes^106–108^. Repurposing DNP and the development of next-generation mild mitochondrial uncouplers that safely modulate mitochondrial bioenergetics may represent a promising therapeutic strategy for acute and chronic neurodegenerative and neuromuscular disorders, particularly at low, body-weight–neutral doses^106^.

In the context of blast TBI, our group demonstrated that repeated mild blast exposures produce persistent mitochondrial dysfunction, characterized by reduced ATP production and elevated oxidative stress^51^. Treatment with a mild mitochondrial uncoupling prodrug restored membrane potential, decreased ROS levels, and improved behavioral outcomes, highlighting the therapeutic potential of controlled mitochondrial uncoupling in blast TBI^51^. Preclinical studies aimed at targeting mitochondrial dysfunction after TBI have proven successful in the neuroprotective effects of mitochondrial uncouplers^109,110^. These interventions may also mitigate broader metabolic deficits observed in metabolomic profiling, including reductions in acetyl-CoA availability and suppression of TCA cycle intermediates. By alleviating oxidative stress, preserving acetyl-CoA availability, and stabilizing mitochondrial membrane potential, MP201 can be used at low dosing as a therapeutic approach aimed at restoring mitochondrial quality control and bioenergetics^111^.

Other next-generation uncouplers have been explored in other preclinical disease models, expanding the repertoire of potential therapeutics. BAM15, tested primarily in metabolic disease models, is orally bioavailable, enhances nutrient oxidation, improves insulin sensitivity, and reduces oxidative stress and inflammation^112^. Niclosamide ethanolamine also acts as a mild mitochondrial uncoupler at nanomolar concentrations, improving energy metabolism, limiting ROS accumulation, and supporting metabolic resilience^113^. Together with MP201, these agents demonstrate the potential of controlled mitochondrial uncoupling as a strategy to restore bioenergetics and protect against secondary injury in blast TBI.

### Mitochondrial biogenesis: enhancing mitochondrial quality control

Mitochondrial biogenesis (MB) is a critical adaptive process for maintaining mitochondrial function and neuronal health, particularly following TBI. Activation of PGC-1α, a master regulator of MB, drives transcription of nuclear-encoded mitochondrial genes, including ATP synthase, cytochrome c, and mitochondrial transcription factor A (TFAM)^114^. Sirtuin-activating compounds, such as resveratrol, stimulate the SIRT1–PGC-1α axis, thereby enhancing mitochondrial biogenesis, improving energy metabolism, and reducing oxidative stress and apoptosis in rodent TBI models^115^. Peroxisome proliferator-activated receptor gamma (PPARγ) has been shown to rapidly increase mitochondrial function via PGC-1α and lead to reductions in oxidative damage^116,117^. These effects have been demonstrated in TBI models, where pioglitazone contributes to improved bioenergetic function and neuroprotection^83,118,119^. More broadly, pioglitazone exemplifies how pharmacological engagement of mitochondrial regulatory proteins can rescue bioenergetic failure after injury and shift cellular outcomes toward recovery.

Resveratrol, including mitochondria-targeted derivatives functionalized with triphenylphosphine, has demonstrated efficacy across multiple CNS models. In Alzheimer’s disease, resveratrol improves synaptic integrity and reduces oxidative stress^120^, while in a murine thoracic blast exposure model, it ameliorates cognitive and emotional deficits, reduces ROS and endoplasmic reticulum stress, and enhances Nrf2 signaling while suppressing NF-κB activation^121^. Preclinical studies of resveratrol have been studied in TBI and indicate favorable promising results, supporting translational potential^122–125^.

Other pharmacologic activators of MB include formoterol, a β₂-adrenoreceptor agonist that increases mitochondrial DNA copy number, enhances mitochondrial respiration, and improves cognitive outcomes in rodent TBI models via the Gβγ-Akt-eNOS-sGC pathway^126^. Furthermore, formoterol therapeutic relevance extends beyond TBI; in a preclinical spinal cord injury model systemic formoterol administration improved mitochondrial content, enhanced respiratory capacity, and supported locomotor recovery, underscoring potential for a β₂-agonist as a broader neurotherapeutic strategy^127^.

### Manipulating mitochondrial dynamics

Blast TBI disrupts mitochondrial quality control, characterized by excessive fission, impaired fusion, and defective mitophagy. These changes lead to the accumulation of damaged organelles, elevated reactive oxygen species (ROS), and progressive bioenergetic decline, ultimately contributing to neuronal dysfunction and cell death. Therapeutic strategies targeting these pathways aim to restore mitochondrial network integrity, enhance organelle turnover, and preserve neuronal viability.

Excessive mitochondrial fission is a symptom of TBI-induced mitochondrial dysfunction, driving mitochondrial fragmentation, ROS production, and apoptosis. Pharmacologic inhibition of fission has demonstrated neuroprotective effects in preclinical models. Mdivi-1, a selective Drp1 inhibitor, preserves mitochondrial morphology, maintains ATP production, and reduces neuronal loss in rodent TBI models^128,129^. Similarly, the TAT-conjugated peptide P110 selectively blocks the Drp1–Fis1 interaction, attenuating pathological fission while sparing normal mitochondrial division. Acute P110 treatment in mice restores mitochondrial morphology, decreases oxidative stress, preserves blood–brain barrier integrity, reduces microglial activation, and improves long-term cognitive outcomes, with effects persisting for up to 17 months post-injury^84^. Importantly, P110’s protective properties extend beyond the CNS, as demonstrated in renal injury models of diabetic nephropathy^130^. By preserving mitochondrial function and curbing Drp1-mediated fragmentation, such interventions mitigate neuronal apoptosis and ameliorate secondary injury post blast TBI.

Mitochondrial fusion is critical for maintaining network connectivity, supporting ATP production, and protecting against apoptosis. Therapeutic promotion of fusion has therefore emerged as a promising strategy to restore mitochondrial dynamics after CNS injury. Several compounds directly target the fusion machinery: for example, celastrol promotes OPA1-dependent fusion and has shown neuroprotective effects in intracerebral hemorrhage^115^, while the small-molecule agonist S89 enhances fusion by specifically activating endogenous MFN1^131^.

### Mitophagy activation

Efficient clearance of damaged mitochondria is essential to maintaining mitochondrial quality control and preventing secondary injury. Urolithin A (UA), a natural compound derived from ellagitannins, induces PINK1/Parkin-dependent mitophagy, reduces oxidative stress, and improves mitochondrial function in preclinical models of TBI and neurodegeneration^132^. In a mouse TBI model, UA reduced blood–brain barrier disruption and neuronal apoptosis, supporting direct CNS relevance^133^.

Spermidine, an endogenous polyamine and dietary compound, is a well-validated autophagy/mitophagy enhancer (AMPK–mTOR–ULK1 axis) with brain penetration. In a closed-head injury mouse model, spermidine improved neurological recovery, reduced BBB damage and apoptosis, and upregulated autophagy markers; convergent work across aging models shows improved mitochondrial quality control, supporting its candidacy as a low-toxicity, orally feasible adjunct^134^. Spermidine studies also show neuroprotective effects in an experimental Parkinson's disease model that are mediated through its antioxidant and anti-inflammatory properties^135^.

### Metabolic activators

In addition to its role in mitochondrial biogenesis, pioglitazone, a member of the thiazolidinedione drug class originally designed as a PPARγ agonist, has also been shown to interact with mitoNEET, a redox-active, pH-sensitive iron–sulfur cluster protein located on the outer mitochondrial membrane^136^. MitoNEET plays a critical role in regulating mitochondrial electron transport chain function^137^.

Sildenafil, a phosphodiesterase-5 (PDE5) inhibitor, has emerged as a potential therapeutic strategy in neurological disorders, including blast TBI, by increasing cGMP levels and activating protein kinase G^138^. Through this pathway, sildenafil enhances neurogenesis, synaptic plasticity via PI3K/Akt signaling, and mitochondrial bioenergetics through nitric oxide-mediated mechanisms that promote mitochondrial biogenesis^139^ and vasodilation^140^. Preclinical studies demonstrate that sildenafil can protect neuronal cells from mitochondrial toxicity and improve overall cellular resilience^141^. Moreover, recent work in mitochondrial diseases such as Leigh syndrome highlights sildenafil’s broader potential to rescue mitochondrial dysfunction, supporting its candidacy as a mitochondria-targeted therapeutic in blast TBI^142^.

Nicotinamide and NAD⁺ precursors have shown promise in activating mitochondrial biogenesis pathways, though further research is needed to establish efficacy in central nervous system (CNS) injury contexts. One such compound, P7C3-A20, has demonstrated neuroprotective effects by activating nicotinamide phosphoribosyl transferase (NAMPT), the rate-limiting enzyme in the NAD⁺ salvage pathway^143^. This activation enhances NAD⁺ synthesis, which is crucial for cellular energy metabolism and mitochondrial function.

### Mitochondrial transplantation

Mitochondrial transplantation involves the delivery of functional, respiratory-competent mitochondria to injured neural tissue, where they integrate into neurons, astrocytes, and glia to restore membrane potential, reduce ROS accumulation, and improve ATP production. Preclinical studies highlight multiple strategies for sourcing and delivering mitochondria. Mitochondria isolated from human umbilical cord–derived mesenchymal stem cells represent a promising donor population that can promote neuronal repair, improve motor function, and attenuate glial activation following experimental TBI^144^. MSC-derived exosomes also provide an alternative route for mitochondrial transfer, as they can deliver intact organelles or mitochondrial components to recipient cells, activating mitophagy and enhancing neuronal survival^144^. Astrocyte-derived extracellular vesicles containing mitochondria may be an avenue for brain-specific delivery^72^. To further improve delivery efficiency, hydrogel-based carriers such as methylcellulose or hyaluronic acid formulations have been developed to retain transplanted mitochondria at the spinal cord injury site, protect them from degradation, and enable gradual release^145^.

Although mitochondrial transplantation demonstrates neuroprotective effects in preclinical models, translation to the clinic remains challenging. Key barriers include optimizing mitochondrial isolation and quality control, refining delivery methods, and establishing long-term efficacy and safety within a regulatory framework^146^. By restoring mitochondrial homeostasis, enhancing bioenergetics, and reinforcing organelle quality control, these approaches may complement pharmacologic interventions such as resveratrol, uncouplers, and PGC-1α activators, offering a multifaceted strategy to improve outcomes after blast TBI. Collectively, these findings underscore a critical principle for blast-related pathology, deliberate manipulation of mitochondrial pathways can alter injury trajectory, reinforcing mitochondria as not only central to pathogenesis but also viable therapeutic targets.

## Conclusion and limitations

**Fig. 3:**
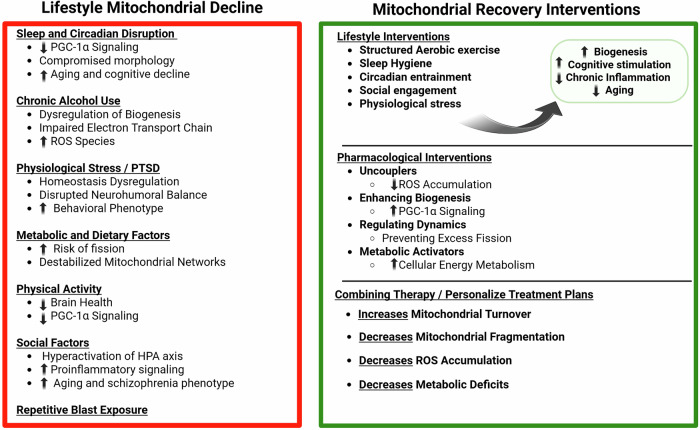
Systemic and environmental modifiers of mitochondrial dysfunction in blast TBI and therapeutic targets. Systemic and environmental modifiers that exacerbate mitochondrial decline in blast TBI (left) and therapeutic interventions aimed at recovery (right). Upward and downward arrows indicate the direction change of functional effects. Created using BioRender.

In conclusion, blast TBI involves overpressure exposure that produces widespread cellular dysfunction in the brain. Blast TBI often produces subtle ultrastructural changes at the cellular and subcellular level, including mitochondrial abnormalities, synaptic disruption, and neurovascular unit compromise, even in the absence of overt tissue lesions. Similar to other TBI subtypes, there are common secondary injury cascades revolving around mitochondrial dysfunction, including disrupted bioenergetics, calcium dysregulation, oxidative stress, and impaired quality control mechanisms such as fission-fusion imbalance and defective mitophagy. The manifestation of these mitochondrial disturbances is further shaped by biological and environmental modifiers, including sleep disturbances, alcohol exposure, and social stressors, which may amplify injury severity or alter recovery trajectories. Such modifiers contribute to the heterogeneous nature of TBI outcomes and underscore the need to consider both injury mechanics and individual susceptibility when evaluating mitochondrial pathology and designing targeted interventions. Therapeutic strategies aimed at restoring mitochondrial health, through modulation of energy metabolism, fusion/fission dynamics, mitophagy, or mitigation of detrimental modifiers, hold promise for improving outcomes. As such, mitochondrial function following blast TBI can be modulated via two complementary approaches: lifestyle-based interventions, such as exercise, diet, and sleep regulation, as detailed in the previous section, and therapeutic strategies, including pharmacological agents and mitochondrial-targeted compounds (Fig. 3).

Together, these insights highlight the central role of mitochondria in mediating blast TBI pathophysiology while contextualizing variability across patients and TBI subtypes. However, much of the current evidence is derived from preclinical models and many studies focus on acute time points without addressing chronic mitochondrial dysfunction or long-term functional consequences. Further, most studies fail to address sex as a biological variable or include both male and female animals. Future research is needed to validate this model of lifespan mitochondrial health after blast TBI using clinically relevant paradigms. These future studies should aim to incorporate the relative contributions of modifiers and optimize targeted mitochondrial interventions for both acute and chronic phases of injury. This review encompasses the theme that mitochondrial dysfunction is a central mediator of neuropathology and neurological deficits following blast TBI, and the trajectory of brain mitochondrial recovery is influenced not only by the primary blast insult but also by systemic and environmental modifiers.

## Data Availability

Data used in this study are available through the Open Data Commons for Traumatic Brain Injury (odc-tbi.org; RRID: SCR_021736)^147^.
